# Research on the effect of professional competency of college physical education teachers on students’ motivation for physical education learning

**DOI:** 10.3389/fpsyg.2025.1620766

**Published:** 2025-10-03

**Authors:** Wanhong Luo, Xu Sun, Zongkai Zhou, Zuguo Tian, Zichao Chen

**Affiliations:** ^1^School of Physical Education, Hunan First Normal University, Changsha, Hunan, China; ^2^School of Physical Education, Hunan University, Changsha, Hunan, China; ^3^School of Physical Education, Sichuan University, Chengdu, Sichuan, China

**Keywords:** college physical education teachers, professional competency, psychological needs satisfaction, physical education learning motivation, self-determination theory

## Abstract

**Introduction:**

This study, based on self-determination theory, constructs a multilevel model to examine the impact of the professional competency of college physical education teachers (PCCPET) on students’ motivation for physical education learning (SMPEL).

**Methods:**

Using sample data from 533 university students, structural equation modeling was applied to explore the influence of physical education teachers’ professional competency on students’ motivation.

**Results:**

The results indicate that the professional competency of college physical education teachers (PCCPET) has a significant positive effect on students’ motivation for physical education learning (SMPEL). Additionally, teacher professional competency indirectly affects students’ motivation through satisfying students’ psychological needs satisfaction for physical education learning (PNSPEL), and this mediating effect is statistically significant. Finally, gender plays a significant moderating role between the professional competency of college physical education teachers (PCCPET) and students’ motivation for physical education learning (SMPEL), with female students being more positively influenced by teacher professional competency.

**Discussion:**

Therefore, in future teaching practices, efforts should be made to enhance the professional competency of physical education teachers and ensure that teachers provide tailored instruction based on students’ gender to effectively stimulate students’ motivation for physical education learning.

## 1 Introduction

Physical education plays a key role not only in improving students’ physical fitness in higher education but also in promoting their mental health and social adaptation (Bull et al., 2020). As global attention to healthy lifestyles and public health continues to increase, university students’ participation in physical activities and sports motivation has become an important focus for teachers when designing curricula and arranging teaching strategies (Keating et al., 2005; Sallis et al., 2016). However, the current level of sports participation and learning motivation among university students remains low, resulting in poor outcomes in physical education and hindering students’ holistic development (Chen et al., 2008; Keating et al., 2005; Lonsdale et al., 2019). According to a report by Strain et al. (2024), 31% of adults and 80% of adolescents fail to meet the recommended levels of physical activity—a phenomenon particularly prevalent among university students. Therefore, enhancing university students’ sports learning motivation has become an urgent issue in physical education research and practice.

Teacher professional competency is considered a key factor influencing educational quality and student learning outcomes (Darling-Hammond, 2000; Shulman, 1987), encompassing subject knowledge, teaching ability, professional ethics, and personality traits (Caena, 2011; Evans, 2011; Hattie, 2008; Özdemir et al., 2019). In the field of physical education, these competencies are particularly crucial, as teachers’ instructional styles, classroom atmosphere, and feedback practices directly influence students’ learning motivation and engagement (Behzadnia et al., 2018; Cheon et al., 2018; Tian and Shen, 2023). Among the many potential determinants of student motivation—such as peer support, family background, or facility accessibility—teacher competency represents the most proximal and policy-malleable factor that universities can directly enhance through preservice preparation and in-service professional development. Qualified teachers implement teaching practices that support autonomy and are well-structured, provide feedback that enhances abilities, and foster emotional connections. This approach meets students’ fundamental psychological needs and stimulates intrinsic motivation. Recent studies confirm that students demonstrate the most adaptive motivational profiles when they perceive their PE teachers as highly autonomy-supportive and structuring but low in controlling behaviors (Fierro-Suero et al., 2024), and intervention trials show that training teachers in autonomy-supportive strategies causally increases students’ need satisfaction, social functioning, and physical activity engagement (Cheon et al., 2022, 2023; Sturm et al., 2021). Systematic reviews further highlight that need-supportive teaching is the proximal driver of autonomous motivation and related outcomes in PE (Vasconcellos et al., 2020), while research on teacher learning emphasizes that these competencies can be deliberately cultivated through professional development (Reeve and Cheon, 2024). In summary, existing research clearly demonstrates that teachers’ professional competence exerts a stronger influence on student learning motivation than other research variables.

Self-determination theory (SDT) posits that an individual’s intrinsic motivation is influenced by the degree to which basic psychological needs—autonomy, competence, and relatedness—are satisfied (Ryan and Deci, 2000; Ryan, 2017). Teachers’ supportive behaviors and teaching methods can promote the satisfaction of these needs, thereby enhancing students’ intrinsic motivation (Cheon et al., 2012; Escriva-Boulley et al., 2018; Jang et al., 2016). Therefore, in physical education, the professional competency of college physical education teachers (PCCPET) not only influences teaching quality, but it also affects students’ learning experience, health behaviors, and future sports participation (García-Ceberino et al., 2022; Luo et al., 2020; Morgan and Hansen, 2008). Conversely, highly competent physical education teachers can create a positive learning environment that fosters students’ intrinsic motivation and positive attitudes toward physical education (Chu et al., 2022; Ferraz et al., 2021; Ginanjar et al., 2021). While participation and learning outcomes are often employed as outcome indicators in PE research, this study focuses exclusively on motivation for physical education learning as the sole dependent variable. This decision is both theoretically justified and empirically supported: motivation operates as a proximal predictor of behavioral engagement and achievement, and serves as a mediating mechanism linking teacher competency to educational outcomes (Eccles and Wigfield, 2020; Reeve and Cheon, 2021). Unlike observable behaviors such as attendance or test scores, which may be shaped by external factors (e.g., institutional scheduling, peer influence), motivation captures a more stable and internalized psychological construct aligned with SDT’s core tenets (Ryan, 2017). Recent research consistently shows that student motivation is a foundational driver for both participation and academic achievement. For example, meta-analyses and large-scale studies indicate that students with higher motivation are more likely to participate actively in class and achieve better academic outcomes (Howard et al., 2021). By centering on motivation, this study provides a more coherent framework to understand how teacher professional competency impacts students’ internal engagement with physical education.

However, the main gaps in the existing research on the impact of the PCCPET on students’ motivation for physical education learning (SMPEL) are two. First, existing research has mainly focused on the primary and secondary school stages, with limited attention to how the PCCPET affects university students’ sports learning motivation (Ntoumanis, 2005; Vasconcellos et al., 2020). Most studies have explored the impact of teachers’ teaching behaviors and professional competency on student motivation in primary and secondary schools (Chen et al., 2012; Morgan and Hansen, 2008), neglecting the particularity of the university environment. University students differ significantly from primary and secondary school students in terms of cognitive abilities, autonomy, and learning needs; this makes it necessary to investigate the impact of the PCCPET on SMPEL in higher education contexts.

Second, research on how the PCCPET affects students’ sports learning motivation—particularly focusing on the relationship between teacher professional competency elements and students’ satisfaction of basic psychological needs—is lacking. SDT has been widely applied to explain students’ learning motivation; however, empirical studies based on SDT in higher education physical education contexts remain limited (Cid et al., 2019; Vasconcellos et al., 2020). Particularly in the context of Chinese higher education, research in this area is even more scarce (Hu et al., 2022; Wenbin, 2020).

Therefore, this study, based on SDT, explores how the PCCPET affects SMPEL by Students’ psychological needs satisfaction for physical education learning (PNSPEL). Specifically, the research questions of this study are as follows: how does the PCCPET affect SMPEL, and what mediating role do basic psychological needs play in this relationship? The contributions of this study are as follows. First, the study enriches and improves theoretical research on the impact of the PCCPET on SMPEL in the field of physical education. Second, based on SDT, it reveals the underlying mechanism through which teacher professional competency influences students’ sports learning motivation, providing a theoretical foundation for future related research. Finally, the empirical results of this study will provide practical guidance for universities in developing training programs and teaching strategies to improve teacher professional competency, better stimulate SMPEL, and promote their health and overall development.

## 2 Theoretical framework

### 2.1 Professional competency of college physical education teachers (PCCPET) and students’ motivation for physical education learning (SMPEL)

The PCCPET is an important research topic in the field of teacher education. According to multiple studies, teacher professional competency includes not only subject knowledge and teaching skills but also teachers’ psychological qualities, values, and emotional attitudes (Darling-Hammond, 2000; Shulman, 1986). Relevant studies have indicated that teacher professional competency can enhance students’ learning motivation through multiple pathways. First, a high level of teacher professional competency can strengthen classroom interaction and emotional engagement, thereby stimulating students’ intrinsic motivation (Ryan and Deci, 2020). Second, teachers’ teaching methods and instructional design are crucial to students’ learning experience. Teachers need not only solid subject knowledge but also a strong understanding of educational psychology to tailor instruction to meet students’ diverse needs (Setyorini and Khuriyah, 2023). Through the combined influence of these competencies, students’ motivation can be effectively improved—especially in physical education—which is a subject with high participation and experiential components (Handoyo et al., 2020). When exploring this hypothesis further, scholars have found that teachers’ teaching behaviors and attitudes not only affect students’ cognitive aspects but also their emotional and motivational changes (Chan et al., 2021). For example, teachers’ positive emotions and effective classroom management strategies can help students maintain higher learning motivation and, to some extent, determine the quality of teaching outcomes (Granero-Gallegos et al., 2019; Reeve, 2012). Based on this theoretical framework, we propose the following hypothesis:

H1: The professional competency of college physical education teachers (PCCPET) significantly positively affects students’ motivation for physical education learning (SMPEL).

### 2.2 Professional competency of college physical education teachers (PCCPET) and students’ psychological needs satisfaction for physical education learning (PNSPEL)

Self-determination theory posits that individuals’ basic psychological needs—including autonomy, competence, and relatedness—significantly impact students’ intrinsic motivation and overall well-being (Deci and Ryan, 2000; Ryan, 2017). In educational contexts, teachers’ behaviors and teaching styles are critical factors influencing students’ satisfaction of these psychological needs (Cheon et al., 2012; Liu et al., 2017). Specifically, the PCCPET—particularly teaching ability, subject knowledge, and emotional support—can significantly influence students’ satisfaction of psychological needs (Behzadnia et al., 2018; Vergara-Torres et al., 2021). On one hand, when teachers demonstrate excellent teaching skills and deep professional knowledge, students are more likely to believe they can succeed in physical education (Tessier et al., 2010). Behzadnia (2021) showed a significant positive correlation between teachers’ professional competency and students’ competence satisfaction. Furthermore, when teachers provide students with appropriate choices and autonomy, students are more likely to experience autonomy satisfaction. Vergara-Torres et al. (2021) indicate that teachers’ use of autonomy-supportive teaching styles can enhance students’ sense of autonomy. On the other hand, if teachers can build positive student–teacher relationships, they can effectively promote students’ relatedness satisfaction, thus improving their learning motivation (Liu et al., 2017; Moreno-Casado et al., 2023). A relevant systematic review (Guo et al., 2023) emphasizes the importance of teacher support in facilitating students’ psychological needs satisfaction and positive learning outcomes. Furthermore, the PCCPET also includes attention to students’ mental health, which is essential for meeting students’ psychological needs (Yan et al., 2022). This indicates that when teachers can identify and address students’ emotional and psychological needs, students’ sense of well-being and satisfaction will be effectively enhanced (Tian and Shen, 2023). Based on these theories and empirical studies, we propose the following hypothesis:

H2: The Professional competency of college physical education teachers (PCCPET) positively influences students’ psychological needs satisfaction for physical education learning (PNSPEL).

### 2.3 Students’ psychological needs satisfaction for physical education learning (PNSPEL) and learning motivation: the mediating effect

In physical education, satisfying students’ psychological needs plays a crucial role in their learning motivation (Curran and Standage, 2017). Therefore, exploring how the satisfaction of students’ psychological needs affects their motivation for physical education learning, as well as the mediating effect it has between teacher professional competency and motivation, has become an important topic in educational research. Students’ psychological needs include autonomy, competence, and relatedness, and satisfying these needs is considered a key factor in enhancing students’ intrinsic motivation. In physical education, fulfilling these basic psychological needs can significantly increase students’ engagement and persistence in physical activities. For instance, García-Ceberino et al. (2022) found that satisfying students’ basic psychological needs in physical education classes could boost their participation, thereby enhancing their exercise compliance. Similarly, Burgueño et al. (2018) found that meeting students’ psychological needs significantly improved their motivation in physical education classes, further encouraging them to continue participating in physical activities outside of class. Additionally, learning motivation is not only directly influenced by the satisfaction of basic psychological needs but also moderated by indirect factors. For example, Behzadnia (2021) found that students’ satisfaction of psychological needs affects their intentions toward physical activities and, by enhancing self-determined motivation, further impacts learning outcomes. Particularly, how teachers satisfy students’ basic psychological needs in the classroom becomes a critical factor in determining students’ learning motivation (Behzadnia et al., 2018).

H3: Students’ psychological needs satisfaction for physical education learning (PNSPEL) significantly positively affects students’ motivation for physical education learning (SMPEL).

Within the relationship between teacher professional competency and SMPEL, psychological needs satisfaction may play an important mediating role. Teachers’ educational approaches and communication styles can effectively meet students’ basic psychological needs, thus influencing students’ learning motivation. Franco and Coterón (2017) found, in an experimental study, that by supporting the satisfaction of students’ basic psychological needs, physical education interventions could enhance their intrinsic motivation and intentions to participate in exercise. This result suggests that teachers not only need good teaching ability, but they should also create a supportive learning environment to satisfy students’ psychological needs, thereby promoting their learning motivation. Further studies have indicated that the mediating effect of psychological needs satisfaction is universal across different cultural contexts. Leo et al. (2022) demonstrated that by meeting students’ psychological needs, teachers’ behaviors indirectly affect students’ learning participation and motivation. In particular, in physical education, teachers can satisfy students’ psychological needs by supporting autonomy, providing feedback and encouragement, and establishing emotional connections, thereby promoting their learning motivation and intentions to engage in physical activities (Tessier et al., 2010).

Therefore, based on SDT, we infer that students’ psychological needs satisfaction mediates the relationship between teacher professional competency and SMPEL. Through effective teaching behaviors that meet students’ psychological needs, teachers can stimulate students’ intrinsic motivation and positively impact their sports learning.

H4: Students’ psychological needs satisfaction for physical education learning (PNSPEL) mediates the relationship between the professional competency of college physical education teachers (PCCPET) and students’ motivation for physical education learning (SMPEL).

### 2.4 Moderating role of students’ gender

**FIGURE 1:**
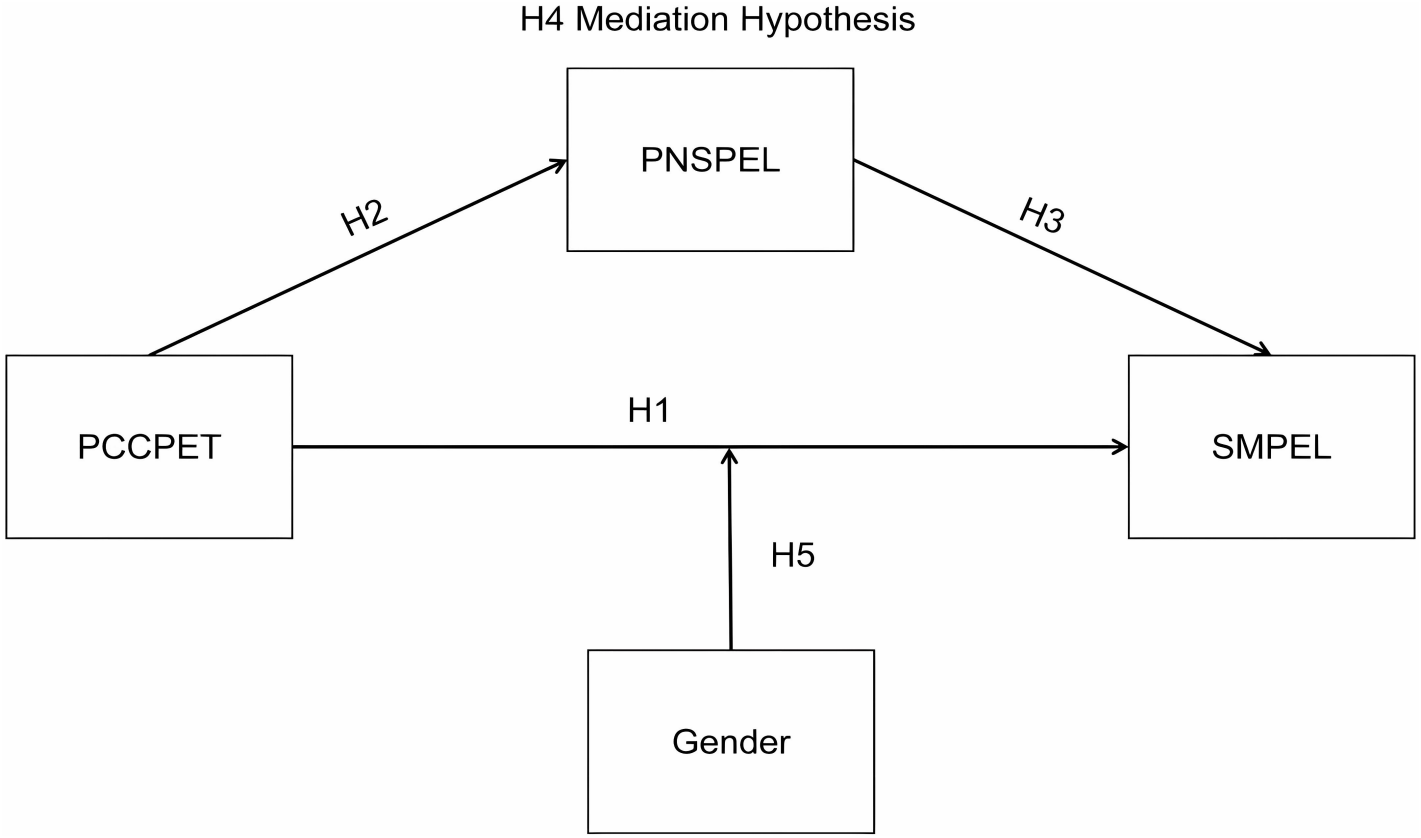
Research model.

The role of students’ gender in physical education has long been an important topic in educational research. Gender factors not only affect participation in physical activities, but they also influence students’ motivation and emotional responses in physical education classes (Laxdal and Giske, 2020). Male and female students are driven by different sources of motivation in physical activities, with males often more motivated by competition and challenging tasks, while females tend to rely more on social interactions and team cooperation (Abós et al., 2021). This gender difference may lead to distinct learning experiences and motivational outcomes in physical education classes, thereby affecting students’ attitudes and participation in the classroom. For example, Kirch et al. (2021) found that gender significantly affects students’ perceptions of teachers’ teaching styles, which in turn moderates students’ learning motivation. Male students tend to respond more positively to authoritative and controlling teaching methods, while female students may rely more on supportive and autonomy-promoting teaching styles. Similarly, regarding teacher professional competency, different effects may arise depending on students’ gender. Van Doren et al. (2021) found, through multilevel analysis, that the interaction between teachers’ teaching styles and students’ gender moderates students’ motivation and participation in physical activities. On one hand, for male students, teachers’ physical education knowledge and high standards may significantly improve their motivation to participate; on the other hand, female students may need more emotional support and psychological safety from teachers in the classroom to better stimulate their motivation and interest in learning. Based on these gender differences, we hypothesize the moderating effect of students’ gender on the relationship between teacher professional competency and SMPEL as follows (see Figure 1):

H5: Students’ gender moderates the relationship between the professional competency of college physical education teachers (PCCPET) and students’ motivation for physical education learning (SMPEL).

## 3 Methodology

### 3.1 Research subjects

This study recruited undergraduate and postgraduate students majoring in physical education from ten higher education institutions in China, comprising five sports universities and five normal universities. To balance scientific rigor with practicality, the study employed a combination of random and convenience sampling. Participants were invited to complete the questionnaire online via the Wenjuanxing platform. This process was entirely voluntary, with informed consent obtained from all respondents, and all responses were collected anonymously. All study procedures were reviewed and approved by the Hunan First Normal University Committee on Science and Technology Ethics (Approval No.: 202501005).

In total, 565 questionnaires were distributed, and 533 valid responses were retained after excluding incomplete or invalid submissions, yielding a valid response rate of 94.34%. The demographic distribution of the sample is summarized in Table 1. Among the respondents, 277 (52.0%) were male and 256 (48.0%) were female, suggesting a balanced gender ratio. With respect to academic grade, the sample included 124 freshmen (23.3%), 154 sophomores (28.9%), 124 juniors (23.3%), 101 seniors (18.9%), and 30 graduate students (5.6%).

**TABLE 1:** Sample description.

Category	n	%
**Gender**		
Male	277	52.00
Female	256	48.00
**Grade**		
Freshman	124	23.30
Sophomore	154	28.90
Junior	124	23.30
Senior	101	18.90
Graduate	30	5.60
Total	533	100.00

Subsequently, the questionnaires were randomly divided into two groups: one for exploratory factor analysis (EFA) with 241 responses and another for confirmatory factor analysis (CFA) with 292 responses, to cross-validate the questionnaire scale.

### 3.2 Questionnaire survey method

#### 3.2.1 Translation process

The scales that required revision were translated and compiled by two graduate students majoring in English, a graduate student in psychology, and two PhD students in physical education. Based on a comparison of the two translated versions, an initial version of the questionnaire was developed. The initial questionnaire was then reviewed by a physical education professor with overseas study experience. After the expert’s feedback was considered, revisions were made by removing or modifying items that were inconsistent or showed substantial differences. The final version of the questionnaire was created based on the expert’s suggestions and the specific context of China.

#### 3.2.2 Reference standards

(1) Exploratory factor analysis (EFA): EFA is designed for situations where the relationships between observed variables and latent factors are unknown or uncertain. The analysis is conducted in an exploratory mode to determine how and to what extent the observed variables are related to the latent factors.

(2) Confirmatory factor analysis (CFA): CFA, which is developed based on EFA, is used in structural equation modeling to test the degree of fit between a hypothesized measurement model (either based on theory or derived from EFA) and the data. The model fit criteria for this study are shown in Table 2.

**TABLE 2:** Model fit indicators and standard criteria.

Indicators	Standard criteria
χ^2^/df	<3
GFI	>0.90
AGFI	>0.90
NFI	>0.90
IFI	>0.90
TLI	>0.90
CFI	>0.90
RFI	>0.90
RMSEA	<0.08
SRMR	<0.08

#### 3.2.3 Questionnaire scale design

To facilitate students in providing more descriptive responses and reduce ambiguity while capturing their true opinions, this study employed a 7-point Likert scale (1 = “strongly disagree” and 7 = “strongly agree”). Compared to a 5-point Likert scale, a 7-point Likert scale provides higher accuracy in participant responses (Joshi et al., 2015) and reduces the tendency of respondents feeling limited owing to fewer response options (Preston and Colman, 2000), thus resulting in more precise and reliable data. However, the response options used should not be too many as this may negatively affect the quality of the questionnaire (Castro-González et al., 2019).

Based on this, we designed the scale measuring the impact of the PCCPET on SMPEL was designed. The scale consists of three dimensions: PCCPET, PNSPEL, and SMPEL, with 29 items. All scales were expert-reviewed, pilot-tested, and will undergo independent reliability and validity analyses in subsequent procedures. The specific scale design is as follows:

(1) The professional competency of college physical education teachers (PCCPET). The design was guided by Arikunto et al. (2023), whose scale assessed pedagogical and professional competencies. Building on this, we added research and ethical competencies to reflect higher education responsibilities, resulting in a 12-item, four-dimension scale.

(2) Students’ psychological needs satisfaction for physical education learning (PNSPEL). Adapted from the Basic Psychological Needs Satisfaction in General Scale (Johnston and Finney, 2010), the scale retained the autonomy, competence, and relatedness structure, with nine items revised for the PE learning context.

(3) Students’ motivation for physical education learning (SMPEL). Based on the Academic Self-Regulation Questionnaire (Ryan and Connell, 1989), the scale captured external, introjected, identified, and intrinsic regulation, refined to eight PE-specific items.

### 3.3 Data processing

SPSS 26.0 was used for EFA on 241 data samples to clarify the factor structure of the questionnaire scale. Subsequently, AMOS 24.0 was used for CFA on 292 data samples to perform cross-validation of the scale and ensure the reliability and validity of the questionnaire. In addition, Pearson correlation analyses were conducted to examine the associations among teacher professional competency, psychological needs satisfaction, and student motivation, as these variables were measured on continuous scales and the large sample size justified the use of parametric tests. Finally, structural equation modeling (SEM) was employed to validate the impact of the PCCPET on SMPEL.

#### 3.3.1 Exploratory factor analysis

The EFA results showed that the KMO index was 0.949, and the Bartlett’s Test of Sphericity was highly significant (*P* < 0.001). Principal axis factoring with the promax rotation method (Kappa = 4) was used to extract factors. The eigenvalue–greater-than-1 criterion and scree plot both supported a three-factor solution, which was consistent with the hypothesized structure. The initial eigenvalues of the first three factors were 9.633, 4.987, and 3.400. After extraction and rotation, the three factors jointly explained 57.80% of the variance, indicating that the solution was both statistically sound and theoretically interpretable. Moreover, all item loadings exceeded 0.70 on their intended factors, with no substantial cross-loadings observed, providing strong evidence of convergent and structural validity for the scale (see Table 3).

**TABLE 3:** Results of exploratory factor analysis during oblique rotations.

Items	Factors loading			Common factor variance (extracted)	Initial eigenvalues	Cumulative percentage of variance/%
	PCCPET	PNSPEL	SMPEL			
Q1	0.709			0.569	9.633	31.768%
Q2	0.746			0.581		
Q3	0.752			0.560		
Q4	0.828			0.674		
Q5	0.736			0.529		
Q6	0.755			0.508		
Q7	0.789			0.575		
Q8	0.777			0.590		
Q9	0.702			0.596		
Q10	0.702			0.544		
Q11	0.784			0.587		
Q12	0.751			0.585		
Q13		0.721		0.541	4.987	47.509%
Q14		0.740		0.546		
Q15		0.807		0.615		
Q16		0.765		0.602		
Q17		0.746		0.574		
Q18		0.730		0.558		
Q19		0.747		0.534		
Q20		0.770		0.599		
Q21		0.726		0.533		
Q22			0.765	0.627	3.400	57.804%
Q23			0.747	0.563		
Q24			0.804	0.620		
Q25			0.816	0.647		
Q26			0.760	0.573		
Q27			0.713	0.557		
Q28			0.762	0.594		
Q29			0.784	0.591		

#### 3.3.2 Confirmatory factor analysis (CFA)

**FIGURE 2:**
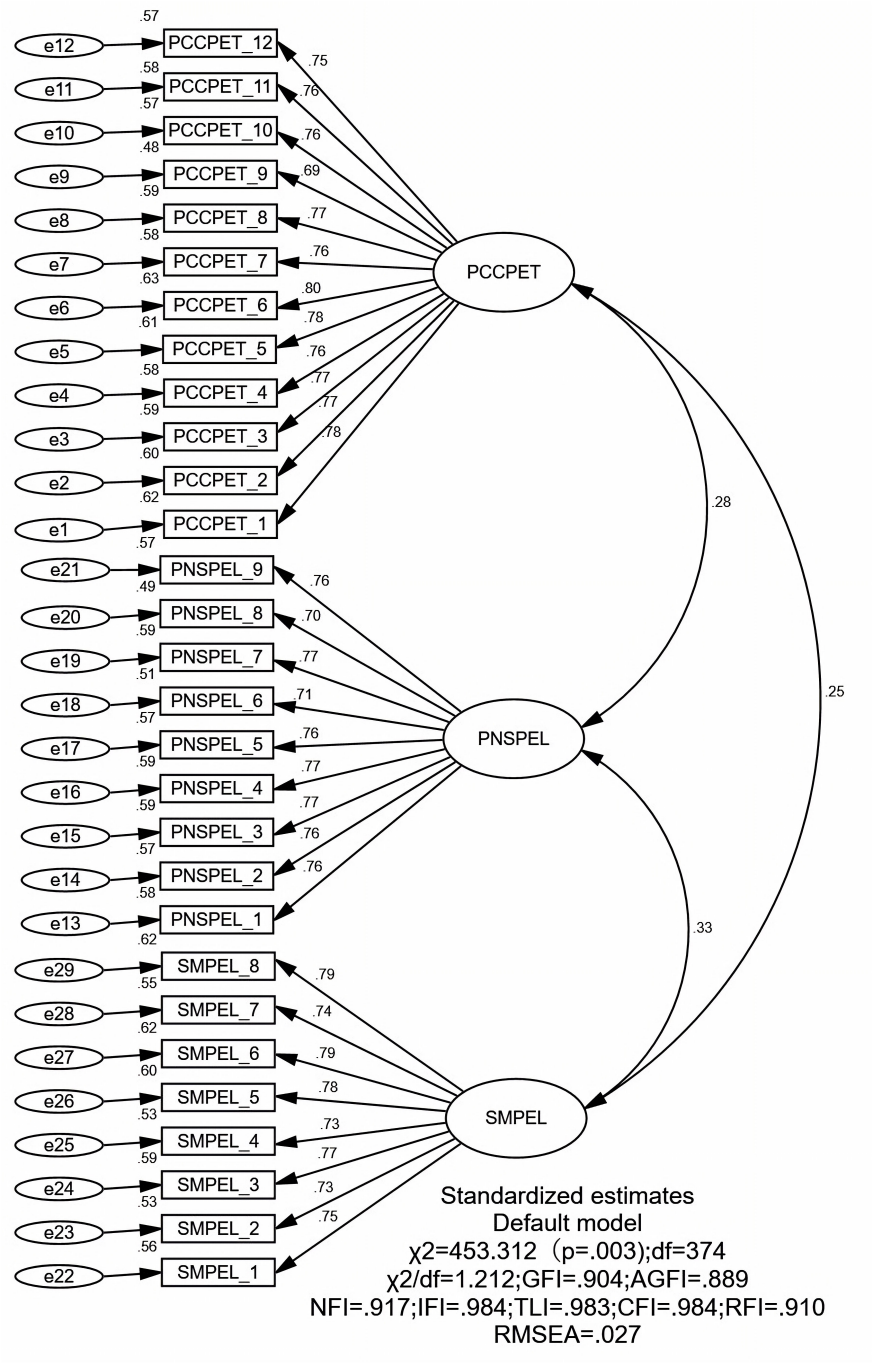
Results of confirmatory factor analysis.

Based on the results from the EFA, CFA was conducted using the other half of the sample data. The results showed that the cross-validation was effective, and the model fit indices were satisfactory (see Figure 2). The fit indices were as follows: Chi-square/degrees of freedom (χ^2^/df) = 1.212, GFI = 0.904, AGFI = 0.889, NFI = 0.917, IFI = 0.984, TLI = 0.983, CFI = 0.984, RFI = 0.910, RMSEA = 0.027, and SRMR = 0.038. These results suggest that the scale’s factor structure is stable and suitable for use as measurement tool in this study.

#### 3.3.3 Reliability and validity testing

The reliability of the instruments was examined through multiple indicators, including Cronbach’s alpha, split-half reliability, composite reliability (CR), and average variance extracted (AVE). As presented in Table 4, all three core subscales—PCCPET, PNSPEL, and SMPEL—achieved Cronbach’s alpha coefficients above 0.90 (0.944, 0.920, and 0.915, respectively). The total questionnaire also demonstrated a high level of internal consistency (α = 0.924), providing strong evidence that the items within each construct coherently measure the intended latent factors. Split-half reliability coefficients further corroborated this stability, with values ranging from 0.906 to 0.941 for the subscales and 0.743 for the overall instrument, which remain within acceptable thresholds for educational and psychological measurement. In addition, the CR values for the three subscales all exceeded 0.90, indicating robust construct reliability and suggesting that the variance captured by the latent constructs was substantially greater than the variance attributable to measurement error. The AVE values ranged from 0.563 to 0.583, surpassing the recommended cutoff of 0.50 (Fornell and Larcker, 1981), thereby confirming adequate convergent validity. Taken together, these results demonstrate that the scales used in this study exhibit strong internal consistency, structural validity, and construct reliability.

**TABLE 4:** Reliability testing.

Indicators	PCCPET	PNSPEL	SMPEL	Total questionnaire
Cronbach α	0.944	0.920	0.915	0.924
Split-half reliability	0.941	0.906	0.912	0.743
CR	0.944	0.920	0.915	
AVE	0.583	0.563	0.575	

#### 3.3.4 Common method bias test

To minimize common method bias in the survey process, this study adopted corresponding measures in both questionnaire design and statistical analysis. First, the questionnaire was designed to be anonymous, and participants were informed that the results were for academic research purposes only, with no right or wrong answers, to reduce social desirability bias (Bergen and Labonté, 2020). Second, Harman’s Single Factor Test was used to examine common method bias. The results showed three factors with eigenvalues greater than 1, and the largest factor explained less than 40% of the variance (Podsakoff et al., 2003), which indicates that common method bias in this study was not significant.

## 4 Results

### 4.1 Descriptive statistics and correlation analysis of key variables

Descriptive statistics and Pearson correlations for the main study variables are presented in Table 5. PCCPET was significantly and positively associated with both PNSPEL (*r* = 0.262, *p* < 0.001) and SMPEL (*r* = 0.232, *p* < 0.001). In addition, PNSPEL was positively correlated with SMPEL (*r* = 0.308, *p* < 0.001). These preliminary associations provide empirical support for the hypothesized relationships and justify subsequent structural equation modeling.

**TABLE 5:** Correlation matrix and average variance extracted.

Variables	Mean	SD	PCCPET	PNSPEL	SMPEL	95% CI
PCCPET	4.295	1.248	1			[4.152, 4.438]
PNSPEL	4.257	1.238	0.262**	1		[4.115, 4.399]
SMPEL	4.224	1.256	0.232**	0.308**	1	[4.080, 4.368]

^**^Refers to *p* < 0.01.

To further aid interpretation of the mean scores, 95% confidence intervals (CIs) were computed for each construct mean using the formula M ± 1.96 × (SD/*n*), where M is the sample mean, SD is the standard deviation, and n is the sample size. This procedure allowed us to determine whether the observed means were significantly higher than the theoretical midpoint of the 7-point scale. On the 7-point scale (midpoint = 4), the mean levels of teacher professional competency (PCCPET; *M* = 4.295, 95% CI [4.152, 4.438]), psychological needs satisfaction (PNSPEL; *M* = 4.257, 95% CI [4.115, 4.399]), and student motivation for PE learning (SMPEL; *M* = 4.224, 95% CI [4.080, 4.368]) were all statistically above the midpoint. This pattern underscores the potential value of targeted, autonomy-supportive pedagogical practices and professional development to shift these perceptions from moderate toward moderately high levels in the higher-education PE context.

### 4.2 Hypothesis testing

#### 4.2.1 Direct effects test

**FIGURE 3:**
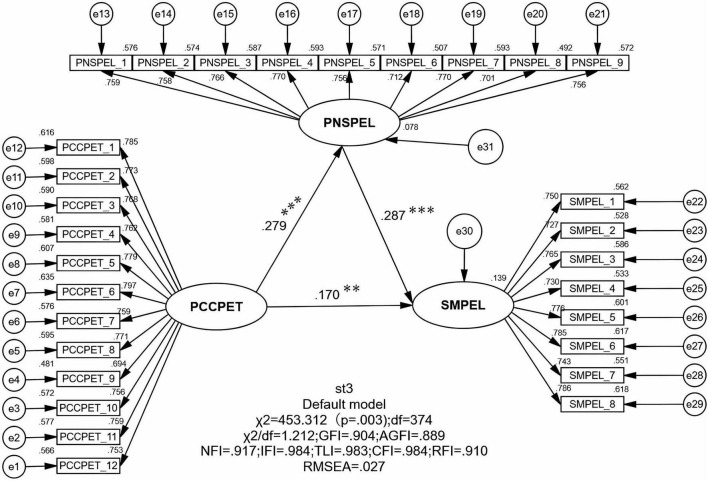
Structural equation model.

According to the results of the structural equation model in this study (Table 6), the standardized path coefficients for the research hypotheses, ordered from largest to smallest, are as follows: PNSPEL → SMPEL, PCCPET → PNSPEL, and PCCPET → SMPEL. The standardized path coefficients for these paths are 0.287, 0.279, and 0.170, respectively, all of which are highly significant (Figure 3). Therefore, the theoretical hypotheses H1, H2, and H3 proposed in this study are supported.

**TABLE 6:** Structural equation modeling path test results.

Paths	Standardized path factors	Unstandardized path factors	S.E.	C.R.	P
H3:PNSPEL→SMPEL	0.287	0.277	0.064	4.345	<0.001
H2:PCCPET→PNSPEL	0.279	0.280	0.064	4.366	<0.001
H1:PCCPET→SMPEL	0.170	0.165	0.061	2.697	0.007

#### 4.2.2 Mediation effect test

This study also calculates the mediation effect of PNSPEL between PCCPET and SMPEL, and tests whether this mediation effect is significant. According to Tofighi and Kelley (2020), the Bootstrap method is more suitable for testing mediation effects compared to the Sobel test, and it provides more precise results. Therefore, the Bootstrap method in AMOS 24.0 was used to test the significance of the mediation effect in this study. The results in Table 7 indicate that the mediation effect of PNSPEL on SMPEL is significant at the 95% confidence interval, excluding 0, with *P* < 0.001, indicating a high level of significance. This suggests that the “PCCPET → PNSPEL → SMPEL” relationship represents a partial mediation model, with the mediation effect accounting for 30.04%, thereby supporting H4.

**TABLE 7:** Bootstrap analysis of mediating effects.

Paths	Unstandardized effect value	SE	*P*	Bias-corrected 95%CI		Percentile 95%CI		Percentage of intermediary effects
				Lower	Upper	Lower	Upper	
Total effect	0.233	0.055	0.001	0.123	0.340	0.125	0.341	30.04%
Direct effect	0.163	0.054	0.003	0.057	0.269	0.058	0.270	
Indirect effect	0.070	0.022	0.001	0.034	0.120	0.031	0.116	

#### 4.2.3 Moderation effect test

To test whether student gender moderates the relationship between the PCCPET and SMPEL, we first centered the variables and dummy-coded gender (0 = male, 1 = female) in SPSS. We then used Model 5 in Process 3.50 to examine the moderation effect of gender on the direct effect of “PCCPET → SMPEL.” The results in Table 8 show that the effect of gender on SMPEL is significant (effect coefficient = 0.323, *P* = 0.025), which indicates that gender significantly influences SMPEL. Additionally, the interaction term between PCCPET and gender is significant (*P* = 0.026), which confirms that gender moderates the relationship between PCCPET and SMPEL, supporting H5.

**TABLE 8:** Moderated mediation effects.

Antecedents	Consequent							
	M(PNSPEL)				Y(SMPEL)			
	Coff.	SE	*P*	95%CI	Coff.	SE	*P*	95%CI
Constant	4.257	0.070	<0.001	[4.119, 4.395]	3.168	0.255	<0.001	[2.666, 3.670]
PCCPET	0.260	0.056	<0.001	[0.149, 0.370]	0.126	0.058	0.032	[0.011, 0.241]
PNSPEL					0.239	0.058	<0.001	[0.124, 0.353]
Gender					0.323	0.143	0.025	[0.042, 0.604]
PCCPET × gender					0.261	0.116	0.026	[0.032, 0.490]
	R^2^ = 0.069, *F* = 21.342, *P* < 0.001				R^2^ = 0.147, *F* = 12.381, *P* < 0.001			

Further analysis shows gender differences in the effects. As shown in Table 9, for female students, the impact of teachers’ professional competence on learning motivation is significantly positive, meaning that the higher the teachers’ professional competence, the stronger the students’ learning motivation (effect value = 0.246, *P* = 0.001). In contrast, for male students, this effect is not significant (effect value = −0.015, *P* = 0.865), which suggests that the motivating effect of teachers’ professional competence is more prominent for female students than for male students.

**TABLE 9:** Moderating effects between genders.

Code	Gender	PCCPET to SMPEL	SE	*t*	*P*	95%CI
0	Male	−0.015	0.09	−0.17	0.865	(−0.192, 0.162)
1	Female	0.246	0.075	3.265	0.001	(0.098, 0.394)

## 5 Discussion

### 5.1 Direct effect of college physical education teachers’ professional competency on students’ motivation for physical education learning

In this study, the PCCPET is defined as teachers’ overall performance in teaching ability, professional knowledge, research skills, and professional ethics. SMPEL is primarily reflected in their interest in the course, participation enthusiasm, and willingness to engage in autonomous learning. The results show a significant positive effect of teachers’ professional competency on SMPEL, which indicates that the higher the teachers’ competency, the stronger the students’ learning motivation. This finding not only aligns with previous studies, but it also emphasizes the key role of teachers’ professional competency in motivating students. Previous research (Lonsdale et al., 2019) has shown that secondary school teachers, by creating more interactive opportunities in class, can significantly enhance students’ enthusiasm for physical activity. Similarly, teaching competence positively influences students’ motivation (Oliver et al., 2018). Yan et al. (2022) pointed out that as teachers’ qualifications and teaching abilities increase, so do their job satisfaction and ability to engage students. Furthermore, Montilla et al. (2023) emphasized that physical education teachers’ ability to incorporate digital technology into their teaching significantly improves students’ motivation for physical learning. This study differs from previous research that has focused on specific dimensions of primary and secondary school teachers’ competency by verifying the direct positive effect of the overall professional competency of college physical education teachers on students’ motivation. These findings support the applicability of self-determination theory in college physical education and provide practical guidance for future teaching practices. This suggests that universities should enhance physical education teachers’ professional development through comprehensive training and assessment mechanisms to better promote students’ motivation.

### 5.2 Direct effect of college physical education teachers’ professional competency on students’ psychological needs satisfaction in physical education learning

The findings of this study align with existing research suggesting that a good classroom atmosphere created by teachers can effectively satisfy students’ psychological needs, thereby enhancing their physical performance and willingness to engage in physical activity (Cid et al., 2019). Similarly, Cheon and Reeve (2015) verified the role of teacher behaviors in fulfilling students’ basic psychological needs, showing that teachers’ strategic support can effectively stimulate students’ learning motivation. Building on these studies, this research further reveals the positive impact of college physical education teachers’ professional competency on students’ psychological needs satisfaction, enriching the understanding of how teachers’ professional competency influences students’ psychological needs. Specifically, the higher the teachers’ professional competency, the more likely they are to stimulate students’ learning motivation by meeting their needs for autonomy, competence, and relatedness. As students’ psychological needs become more diverse in higher education, focusing on satisfying these needs in future physical education practices is essential for teachers. Teachers can achieve this by offering students choices and decision-making power, ensuring that their psychological needs are comprehensively supported.

### 5.3 The impact of students’ psychological needs satisfaction on their motivation for physical education learning

Teachers, by creating an environment conducive to satisfying students’ psychological needs, can effectively enhance students’ engagement and enthusiasm, thus further promoting their motivation for physical education (Vasconcellos et al., 2020). In particular, when students experience autonomy, competence, and belonging in class, they are more likely to maintain a lasting interest and motivation for physical education. This study verifies that the satisfaction of students’ psychological needs in physical education significantly positively affects their learning motivation. Additionally, students’ psychological needs satisfaction plays a partial mediating role in the relationship between teachers’ professional competency and SMPEL. In other words, teachers’ professional competency indirectly enhances students’ motivation by fulfilling their basic psychological needs. This mediation effect further validates the key role of teachers in supportive teaching environments and underscores the importance of teachers’ professional competency in motivating students. These findings not only align with the existing literature (Coterón et al., 2024), but they also deepen the understanding of the relationship between teachers’ professional competency, psychological needs satisfaction, and SMPEL.

### 5.4 The moderating effect of gender on the relationship between college physical education teachers’ professional competency and students’ motivation for physical education learning

In research involving gender as a moderating variable, scholars have generally agreed that gender plays a substantial role in moderating behaviors such as consumer behavior and decision-making (Robledo et al., 2015; Tarhini et al., 2014). However, research on the impact of college physical education teachers’ competency on students’ motivation by gender remains limited. This study shows that female students are more influenced by teachers’ professional competency, indicating that female students’ motivation in physical education is more easily stimulated by teachers’ professional qualities. This could be because female students are more sensitive to teachers’ support, feedback, and encouragement. Female students tend to have higher relationship needs and place more importance on support and recognition in the learning environment (Eccles and Roeser, 2011). Conversely, male students’ motivation for physical education is not influenced by teachers’ professional competency, which suggests that they are less dependent on external feedback and may demonstrate more autonomy in their physical learning.

This gender difference suggests that future teaching practices should incorporate gender sensitivity training in teachers’ professional development. Teachers need to understand the different needs of male and female students and provide personalized feedback based on gender characteristics. By doing so, teachers can maximize opportunities for students to have their psychological needs met, thereby improving their learning motivation.

## 6 Conclusion

### 6.1 Summary of key findings

In conclusion, this study, through a moderated mediation model, explores the impact of college physical education teachers’ professional competency on SMPEL using SDT. The results show that teachers’ professional competency positively influences students’ motivation for physical education by enhancing their psychological needs satisfaction. Moreover, teachers’ competency not only directly affects students’ learning motivation, but it also plays an indirect role by satisfying students’ psychological needs, with students’ motivation acting as a mediator. Gender significantly moderates the relationship between teachers’ professional competency and students’ motivation, with female students being more influenced by teachers’ professional competency than male students. This research provides a new perspective for college physical education teaching and highlights the importance of gender differences in the educational process.

### 6.2 Theoretical and practical implications

This study makes the following four theoretical contributions: First, it deepens the theoretical understanding of SDT within the higher education physical education context. Previous research has predominantly focused on physical education classrooms in primary and secondary schools, with insufficient exploration of how university physical education instructors’ professional competence influences SMPEL. This study thus expands the applicability of SDT in the field of higher education physical education and addresses the existing gap in contextual boundaries within the literature. Second, it reveals the mediating mechanism of psychological need satisfaction between teachers’ professional competence and students’ motivation. By incorporating the mediating variable of psychological need satisfaction in physical education learning, this study enhances the theoretical explanatory power of SDT regarding the intrinsic mechanisms linking university physical education teachers’ professional competence and SMPEL. It also provides new validation pathways for subsequent research. Third, existing research tools are largely confined to general educational settings and lack multidimensional characterizations of university physical education teachers’ professional competencies. This study refines the measurement system within the context of university physical education, providing a more precise operational tool for future assessments of both teachers’ professional competencies and students’ learning motivation in physical education. Fourth, this study found that female SMPEL is more susceptible to the positive influence of teachers’ professional competence, whereas male students’ motivation remains relatively independent of such competence. Consequently, this discovery offers new insights for developing differentiated motivational theories in future research.

The practical value of linking teacher professional competency to students’ motivation in higher-education PE lies in its malleability: elevating what teachers do—in teaching craft, subject expertise, research literacy, and professional ethics—translates into measurable gains in students’ motivational states. First, Universities should therefore operationalize competency as day-to-day need-supportive teaching. In class, this means offering meaningful choices and clear rationales, setting transparent success criteria, sequencing tasks progressively, giving process-focused feedback, and cultivating an inclusive climate—behaviors that satisfy autonomy, competence, and relatedness and, in turn, lift motivation. Second, Professional development ought to be modular and evidence-anchored: micro-teaching with video-based reflection on autonomy-support cues; content refreshers tied to contemporary PE science; practice-oriented research literacy; and ethics-in-practice for fair assessment and feedback. Finally, adopt gender-responsive routines: for many female students, amplify recognition, dialogic feedback, and participatory voice; for many male students, increase mastery-tracking and calibrated challenge without reducing standards. Align hiring, appraisal, and promotion with demonstrated growth in need-supportive teaching, and ensure facilities and scheduling enable differentiated tasks.

### 6.3 Research limitations and prospects

Although this study yielded valuable findings, some limitations remain. First, the design is cross-sectional, which makes it difficult to establish temporal precedence among teacher professional competency, students’ psychological needs satisfaction, and motivation for physical education learning. Future research could employ longitudinal tracking or competency-focused professional development interventions to examine changes over time and to verify the proposed pathway. Second, all focal constructs were measured via same-time student self-reports, which may introduce common-method variance and inflate associations. Subsequent studies should triangulate data using multi-informant sources (e.g., classroom observations with need-support rubrics, teacher self-reports) and low-burden behavioral indicators of engagement, or introduce temporal separation/marker variables to mitigate method bias. Third, the sample comprised PE majors from Chinese universities and was obtained through a combination of random and convenience procedures in clustered settings, therefore, generalizability should be confined to similar higher-education PE contexts. Future work could adopt multi-region probability sampling and multilevel SEM to model class/institution effects and test measurement invariance across subgroups. Fourth, we focused on gender as a moderator variable without incorporating other potential covariates (such as teacher experience, class size, access to facilities, and peer norms), though future research could further refine the design by integrating these covariates.

## Data Availability

The raw data supporting the conclusions of this article will be made available by the authors, without undue reservation.

## References

[B1] Abós Á Burgueño R. García-González L. Sevil-Serrano J. (2021). Influence of internal and external controlling teaching behaviors on students’ motivational outcomes in physical education: Is there a gender difference? *J. Teach. Phys. Educ.* 41 502–512. 10.1123/jtpe.2020-0316

[B2] Arikunto S. Saputra W. N. E. Khoirunnisa D. A. Sawai R. P. (2023). Development and validation of teacher competency perception scale in Indonesia: The Rasch analysis. *J. Profess. Teach. Educ.* 1 11–21. 10.12928/jprotect.v1i1.491

[B3] Behzadnia B. (2021). The relations between students’ causality orientations and teachers’ interpersonal behaviors with students’ basic need satisfaction and frustration, intention to physical activity, and well-being. *Phys. Educ. Sport Pedag.* 26 613–632. 10.1080/17408989.2020.1849085

[B4] Behzadnia B. Adachi P. J. Deci E. L. Mohammadzadeh H. (2018). Associations between students’ perceptions of physical education teachers’ interpersonal styles and students’ wellness, knowledge, performance, and intentions to persist at physical activity: A self-determination theory approach. *Psychol. Sport Exerc.* 39 10–19. 10.1016/j.psychsport.2018.07.003

[B5] Bergen N. Labonté R. (2020). “Everything is perfect, and we have no problems”: Detecting and limiting social desirability bias in qualitative research. *Qual. Health Res.* 30 783–792. 10.1177/1049732319889354 31830860

[B6] Bull F. C. Al-Ansari S. S. Biddle S. Borodulin K. Buman M. P. Cardon G. (2020). World health organization 2020 guidelines on physical activity and sedentary behaviour. *Br. J. Sports Med.* 54 1451–1462. 10.1136/bjsports-2020-102955 33239350 PMC7719906

[B7] Burgueño R. Cueto-Martín B. E. N. Morales-Ortiz E. Silva P. C. Medina-Casaubon J. (2018). Clarifying the influence of sport education on basic psychological need satisfaction in high school students. *Motricidade* 14 48–58. 10.6063/motricidade.13318 40564005

[B8] Caena F. (2011). *Literature review teachers’ core competences: Requirements and development. European commission thematic working group on professional development of teachers.* Brussels: European Commission.

[B9] Castro-González S. Bande B. E. N. Fernández-Ferrín P. Kimura T. (2019). Corporate social responsibility and consumer advocacy behaviors: The importance of emotions and moral virtues. *J. Clean. Prod.* 231 846–855. 10.1016/j.jclepro.2019.05.238

[B10] Chan M. Sharkey J. D. Lawrie S. I. Arch D. A. Nylund-Gibson K. (2021). Elementary school teacher well-being and supportive measures amid COVID-19: An exploratory study. *Sch. Psychol.* 36:533. 10.1037/spq0000441 34292036

[B11] Chen A. Martin R. Ennis C. D. Sun H. (2008). Content specificity of expectancy beliefs and task values in elementary physical education. *Res. Q. Exerc. Sport* 79 195–208. 10.1080/02701367.2008.10599483 18664044 PMC4477638

[B12] Chen S. Chen A. Zhu X. (2012). Are K–12 learners motivated in physical education? A meta-analysis. *Res. Q. Exerc. Sport* 83 36–48. 10.1080/02701367.2012.10599823 22428410

[B13] Cheon S. H. Reeve J. (2015). A classroom-based intervention to help teachers decrease students’ amotivation. *Contemp. Educ. Psychol.* 40 99–111. 10.1016/j.cedpsych.2014.06.004

[B14] Cheon S. H. Reeve J. Marsh H. W. (2023). Autonomy-supportive teaching enhances prosocial and reduces antisocial behavior via classroom climate and psychological needs: A multilevel randomized control intervention. *J. Sport Exerc. Psychol.* 45 26–40. 10.1123/jsep.2021-0337 36634307

[B15] Cheon S. H. Reeve J. Moon I. S. (2012). Experimentally based, longitudinally designed, teacher-focused intervention to help physical education teachers be more autonomy supportive toward their students. *J. Sport Exerc. Psychol.* 34 365–396. 10.1123/jsep.34.3.365 22691399

[B16] Cheon S. H. Reeve J. Ntoumanis N. (2018). A needs-supportive intervention to help PE teachers enhance students’ prosocial behavior and diminish antisocial behavior. *Psychol. Sport Exerc.* 35 74–88. 10.1016/j.psychsport.2017.11.010

[B17] Cheon S. H. Reeve J. Marsh H. W. Song Y.-G. (2022). Intervention-enabled autonomy-supportive teaching improves the PE classroom climate to reduce antisocial behavior. *Psychol. Sport Exerc.* 60:102174. 10.1016/j.psychsport.2022.102174 36634307

[B18] Chu Y. Chen C. Wang G. Su F. (2022). The effect of education model in physical education on student learning behavior. *Front. Psychol.* 13:944507. 10.3389/fpsyg.2022.944507 35874372 PMC9305612

[B19] Cid L. Pires A. Borrego C. Duarte-Mendes P. Teixeira D. S. Moutão J. A. O. M. (2019). Motivational determinants of physical education grades and the intention to practice sport in the future. *PLoS One* 14:e217218. 10.1371/journal.pone.0217218 31120973 PMC6592572

[B20] Coterón J. Fernández-Caballero J. Martín-Hoz L. Franco E. (2024). The interplay of structuring and controlling teaching styles in physical education and its impact on students’ motivation and engagement. *Behav. Sci.* 14:836. 10.3390/bs14090836 39336051 PMC11428318

[B21] Curran T. Standage M. (2017). Psychological needs and the quality of student engagement in physical education: Teachers as key facilitators. *J. Teach. Phys. Educ.* 36 262–276. 10.1123/jtpe.2017-0065

[B22] Darling-Hammond L. (2000). Teacher quality and student achievement. *Educ. Policy Anal. Arch.* 8:1. 10.14507/epaa.v8n1.2000

[B23] Deci E. L. Ryan R. M. (2000). The “what” and “why” of goal pursuits: Human needs and the self-determination of behavior. *Psychol. Inq.* 11 227–268. 10.1207/S15327965PLI110401

[B24] Eccles J. S. Roeser R. W. (2011). Schools as developmental contexts during adolescence. *J. Res. Adolesc.* 21 225–241. 10.1111/j.1532-7795.2010.00725.x

[B25] Eccles J. S. Wigfield A. (2020). From expectancy-value theory to situated expectancy-value theory: A developmental, social cognitive, and sociocultural perspective on motivation. *Contemp. Educ. Psychol.* 61:101859. 10.1016/j.cedpsych.2020.101859

[B26] Escriva-Boulley G. E. R. Tessier D. Ntoumanis N. Sarrazin P. (2018). Need-supportive professional development in elementary school physical education: Effects of a cluster-randomized control trial on teachers’ motivating style and student physical activity. *Sport Exerc. Perform. Psychol.* 7:218. 10.1037/spy0000119

[B27] Evans L. (2011). The ‘shape’ of teacher professionalism in England: Professional standards, performance management, professional development and the changes proposed in the 2010 White Paper. *Br. Educ. Res. J.* 37 851–870. 10.1080/01411926.2011.607231

[B28] Ferraz R. Silva M. Marinho D. A. Neiva H. P. Branquinho L. (2021). Student motivation associated with the practice of individual and team sports in physical education classes. *J. Adv. Sport Phys. Educ.* 4 51–58. 10.36348/jaspe.2021.v04i04.002

[B29] Fierro-Suero S. Van Doren N. De Cocker K. Haerens L. (2024). Towards a refined insight into physical education teachers’ autonomy-supportive, structuring, and controlling style to the importance of student motivation: A person-centered approach. *Phys. Educ. Sport Pedag.* 1–17. 10.1080/17408989.2024.2432307

[B30] Fornell C. Larcker D. F. (1981). Evaluating structural equation models with unobservable variables and measurement error. *J. Market. Res.* 18 39–50. 10.1177/002224378101800104

[B31] Franco E. Coterón J. (2017). The effects of a physical education intervention to support the satisfaction of basic psychological needs on the motivation and intentions to be physically active. *J. Hum. Kinet.* 59:5. 10.1515/hukin-2017-0143 29134044 PMC5680682

[B32] García-Ceberino J. M. Feu S. Gamero M. G. Ibáñez S. J. (2022). Determinant factors of achievement motivation in school physical education. *Children* 9:1366. 10.3390/children9091366 36138675 PMC9497943

[B33] Ginanjar A. Mubarok M. Z. Mudzakir D. O. (2021). College students’ motivation after teaching using sport education season. *Int. J. Hum. Mov. Sports Sci.* 9 1–7. 10.13189/saj.2021.091301

[B34] Granero-Gallegos A. Ruiz-Montero P. J. Baena-Extremera A. Martínez-Molina M. (2019). Effects of motivation, basic psychological needs, and teaching competence on disruptive behaviours in secondary school physical education students. *Int. J. Environ. Res. Public Health* 16:4828. 10.3390/ijerph16234828 31805635 PMC6926537

[B35] Guo Q. Samsudin S. Yang X. Gao J. Ramlan M. A. Abdullah B. (2023). Relationship between perceived teacher support and student engagement in physical education: A systematic review. *Sustainability* 15:6039. 10.3390/su15076039

[B36] Handoyo M. T. Priambodo A. Kumaat N. A. (2020). The relationship of professional competence of physical education sport and health teachers to the implementation of the 2013 curriculum of physical education, sport and health in elementary schools in Tambaksari District. *Br. Int. Hum. Soc. Sci. J.* 2 529–536. 10.33258/biohs.v2i2.255

[B37] Hattie J. (2008). *Visible learning: A synthesis of over 800 meta-analyses relating to achievement.* Milton Park: Routledge, 10.4324/9780203887332

[B38] Howard J. L. Bureau J. Guay F. Chong J. X. Y. Ryan R. M. (2021). Student motivation and associated outcomes: A meta-analysis from self-determination theory. *Perspect. Psychol. Sci.* 16 1300–1323. 10.1177/1745691620966789 33593153

[B39] Hu T. Zhang M. Liu H. Liu J. Pan S. Guo J. (2022). The influence of “small private online course+ flipped classroom” teaching on physical education students’ learning motivation from the perspective of self-determination theory. *Front. Psychol.* 13:938426. 10.3389/fpsyg.2022.938426 36081715 PMC9447416

[B40] Jang H. Kim E. J. Reeve J. (2016). Why students become more engaged or more disengaged during the semester: A self-determination theory dual-process model. *Learn. Instruct.* 43 27–38. 10.1016/j.learninstruc.2016.01.002

[B41] Johnston M. M. Finney S. J. (2010). Measuring basic needs satisfaction: Evaluating previous research and conducting new psychometric evaluations of the basic needs satisfaction in general scale. *Contemp. Educ. Psychol.* 35 280–306. 10.1016/j.cedpsych.2010.04.003

[B42] Joshi A. Kale S. Chandel S. Pal D. K. (2015). Likert scale: Explored and explained. *Br. J. Appl. Sci. Technol.* 7 396–403. 10.9734/BJAST/2015/14975

[B43] Keating X. D. Guan J. Piñero J. E. C. Bridges D. M. (2005). A meta-analysis of college students’ physical activity behaviors. *J. Am. Coll. Health* 54 116–126. 10.3200/JACH.54.2.116-126 16255324

[B44] Kirch A. Schnitzius M. Spengler S. Blaschke S. Mess F. (2021). Knowing students’ characteristics: Opportunities to adapt physical education teaching. *Front. Psychol.* 12:619944. 10.3389/fpsyg.2021.619944 33643149 PMC7907514

[B45] Laxdal A. Giske R. (2020). Gender and the perceived learning environment in upper secondary school physical education. *Sport Educ. Soc.* 25 779–787. 10.1080/13573322.2019.1666360

[B46] Leo F. M. Mouratidis A. Pulido J. J. López-Gajardo M. A. Sánchez-Oliva D. (2022). Perceived teachers’ behavior and students’ engagement in physical education: The mediating role of basic psychological needs and self-determined motivation. *Phys. Educ. Sport Pedag.* 27 59–76. 10.1080/17408989.2020.1850667

[B47] Liu J. Bartholomew K. Chung P. (2017). Perceptions of teachers’ interpersonal styles and well-being and ill-being in secondary school physical education students: The role of need satisfaction and need frustration. *Sch. Ment. Health* 9 360–371. 10.1007/s12310-017-9223-6

[B48] Lonsdale C. Lester A. Owen K. B. White R. L. Peralta L. Kirwan M. (2019). An internet-supported school physical activity intervention in low socioeconomic status communities: Results from the activity and motivation in physical education (AMPED) cluster randomised controlled trial. *Br. J. Sports Med.* 53 341–347. 10.1136/bjsports-2017-097904 28993404

[B49] Luo Y. Lin M. Hsu C. Liao C. Kao C. (2020). The effects of team-game-tournaments application towards learning motivation and motor skills in college physical education. *Sustainability* 12:6147. 10.3390/su12156147

[B50] Montilla V. R. Rodriguez R. Aliazas J. V. Gimpaya R. (2023). Teachers’ pedagogical digital competence as relevant factors on academic motivation and performance in physical education. *Int. J. Sci. Manag. Res.* 6, 45–58. 10.37502/IJSMR.2023.6604

[B51] Moreno-Casado H. E. C. Leo F. M. López-Gajardo M. A. García-Calvo T. A. S. Pulido J. J. (2023). Teachers’ verbal and nonverbal communication, students’ psychological needs, and positive and negative outcomes in physical education. *J. Sport Exerc. Psychol.* 45 269–278. 10.1123/jsep.2022-0240 37666499

[B52] Morgan P. J. Hansen V. (2008). Classroom teachers’ perceptions of the impact of barriers to teaching physical education on the quality of physical education programs. *Res. Q. Exerc. Sport* 79 506–516. 10.1080/02701367.2008.10599517 19177952

[B53] Ntoumanis N. (2005). A prospective study of participation in optional school physical education using a self-determination theory framework. *J. Educ. Psychol.* 97:444. 10.1037/0022-0663.97.3.444

[B54] Oliver K. L. Luguetti C. Aranda R. Nuñez-Enriquez O. Rodriguez A. (2018). ‘Where do I go from here?’: Learning to become activist teachers through a community of practice. *Phys. Educ. Sport Pedag.* 23 150–165. 10.1080/17408989.2017.1350263

[B55] Özdemir T. Y. Demirkol M. Polat H. (2019). Teaching as a professionalism through teachers’ perspective. *Turk. Online J. Q. Inq.* 10 296–320. 10.17569/tojqi.498776

[B56] Podsakoff P. M. MacKenzie S. B. Lee J. Podsakoff N. P. (2003). Common method biases in behavioral research: A critical review of the literature and recommended remedies. *J. Appl. Psychol.* 88:879. 10.1037/0021-9010.88.5.879 14516251

[B57] Preston C. C. Colman A. M. (2000). Optimal number of response categories in rating scales: Reliability, validity, discriminating power, and respondent preferences. *Acta Psychol.* 104 1–15. 10.1016/S0001-6918(99)00050-5 10769936

[B58] Reeve J. (2012). “A self-determination theory perspective on student engagement,” in *Handbook of research on student engagement*, eds Christenson S. L. Reschly A. L. Wylie C. (Berlin: Springer), 149–172. 10.1007/978-1-4614-2018-7_7

[B59] Reeve J. Cheon S. H. (2021). Autonomy-supportive teaching: Its malleability, benefits, and potential to improve educational practice. *Educ. Psychol.* 56 54–77. 10.1080/00461520.2020.1862657

[B60] Reeve J. Cheon S. H. (2024). Learning how to become an autonomy-supportive teacher begins with perspective taking: A randomized control trial and model test. *Teach. Teach. Educ.* 148:104702. 10.1016/j.tate.2024.104702

[B61] Robledo J. E. L. R. Arán M. I. A. V. Sánchez V. M. Molina M. A. N. R. (2015). The moderating role of gender on entrepreneurial intentions: A TPB perspective. *Intangible Cap.* 11 92–117. 10.3926/ic.557

[B62] Ryan R. M. (2017). *Self-determination theory: Basic psychological needs in motivation, development, and wellness.* New York, NY: Guilford Press, 10.7202/1041847ar

[B63] Ryan R. M. Connell J. P. (1989). Perceived locus of causality and internalization: Examining reasons for acting in two domains. *J. Pers. Soc. Psychol.* 57:749. 10.1037/0022-3514.57.5.749 2810024

[B64] Ryan R. M. Deci E. L. (2000). Self-determination theory and the facilitation of intrinsic motivation, social development, and well-being. *Am. Psychol.* 55, 68–78. 10.1037/0003-066X.55.1.68 11392867

[B65] Ryan R. M. Deci E. L. (2020). Intrinsic and extrinsic motivation from a self-determination theory perspective: Definitions, theory, practices, and future directions. *Contemp. Educ. Psychol.* 61 101860. 10.1016/j.cedpsych.2020.101860

[B66] Sallis J. F. Bull F. Guthold R. Heath G. W. Inoue S. Kelly P. (2016). Progress in physical activity over the *Olympic quadrennium*. *Lancet* 388 1325–1336. 10.1016/S0140-6736(16)30581-5 27475270

[B67] Setyorini E. T. Khuriyah K. (2023). The influence of teacher professionalism and creativity on student motivation in Madrasah Ibtidaiyah. *Attadrib* 6 40–47. 10.54069/attadrib.v6i1.374

[B68] Shulman L. S. (1986). Those who understand: Knowledge growth in teaching. *Educ. Res.* 15 4–14. 10.3102/0013189X015002004 38293548

[B69] Shulman L. S. (1987). Knowledge and teaching: Foundations of the new reform. *Harvard Educ. Rev.* 57 1–23. 10.17763/haer.57.1.j463w79r56455411

[B70] Strain T. Flaxman S. Guthold R. Semenova E. Cowan M. Riley L. M. (2024). National, regional, and global trends in insufficient physical activity among adults from 2000 to 2022: A pooled analysis of 507 population-based surveys with 5.7 million participants. *Lancet Glob. Health* 12 e1232–e1243. 10.1016/S2214-109X(24)00150-5 38942042 PMC11254784

[B71] Sturm D. J. Bachner J. Renninger D. Haug S. Demetriou Y. (2021). A cluster randomized trial to evaluate need-supportive teaching in physical education on physical activity of sixth-grade girls: A mixed method study. *Psychol. Sport Exerc.* 54:101902. 10.1016/j.psychsport.2021.101902

[B72] Tarhini A. Hone K. Liu X. (2014). Measuring the moderating effect of gender and age on e-learning acceptance in England: A structural equation modeling approach for an extended technology acceptance model. *J. Educ. Comput. Res.* 51 163–184. 10.2190/EC.51.2.b 22612255

[B73] Tessier D. Sarrazin P. Ntoumanis N. (2010). The effect of an intervention to improve newly qualified teachers’ interpersonal style, students’ motivation and psychological need satisfaction in sport-based physical education. *Contemp. Educ. Psychol.* 35 242–253. 10.1016/j.cedpsych.2010.05.005

[B74] Tian L. Shen J. (2023). The effect of perceived teachers’ interpersonal behavior on students’ learning in physical education: A systematic review. *Front. Psychol.* 14:1233556. 10.3389/fpsyg.2023.1233556 37720632 PMC10499622

[B75] Tofighi D. Kelley K. (2020). Indirect effects in sequential mediation models: Evaluating methods for hypothesis testing and confidence interval formation. *Multivar. Behav. Res.* 55 188–210. 10.1080/00273171.2019.1618545 31179751 PMC6901816

[B76] Van Doren N. De Cocker K. De Clerck T. Vangilbergen A. Vanderlinde R. Haerens L. (2021). The relation between physical education teachers’ (de-)motivating style, students’ motivation, and students’ physical activity: A multilevel approach. *Int. J. Environ. Res. Public Health* 18:7457. 10.3390/ijerph18147457 34299907 PMC8307004

[B77] Vasconcellos D. Parker P. D. Hilland T. Cinelli R. Owen K. B. Kapsal N. (2020). Self-determination theory applied to physical education: A systematic review and meta-analysis. *J. Educ. Psychol.* 112 1444–1469. 10.1037/edu0000420

[B78] Vergara-Torres A. P. Tristán J. E. López-Walle J. M. González-Gallegos A. Pappous A. S. Tomás I. E. S. (2021). Quality of the physical education teacher’s instruction in the perspective of self-determination. *Front. Psychol.* 12:708441. 10.3389/fpsyg.2021.708441 34354649 PMC8330812

[B79] Wenbin L. (2020). Research on factors influencing college students’ initiative in physical learning. *Learning* 2 4–6. 10.25236/FSR.2020.020402

[B80] Yan T. Teo E. W. Lim B. H. Lin B. (2022). Evaluation of competency and job satisfaction by positive human psychology among physical education teachers at the university level: A systematic review. *Front. Psychol.* 13:1084961. 10.3389/fpsyg.2022.1084961 36605263 PMC9810261

